# Correlation of Cognition With Disability and Physical Performance in Patients With Relapsing-Remitting MS

**DOI:** 10.1177/11795735251349716

**Published:** 2025-06-17

**Authors:** Marko Luostarinen, Anne M Portaankorva, Pirjo Urpilainen, Saara Takala, Mika Venojärvi

**Affiliations:** 1 Institute of Biomedicine, Sports and Exercise Medicine, 4344 University of Eastern Finland, Kuopio, Finland; 2 Research Unit of Clinical Medicine, Neurology, 176467 University of Oulu, Finland; 3 MRC, Oulu University Hospital and University of Oulu, Finland; 4 Clinical Neurosciences, Faculty of Medicine, 60655 University of Helsinki, Finland; 5 School of Medicine, 101232 University of Eastern Finland, Kuopio, Finland

**Keywords:** multiple sclerosis, disability, cognition, physical activity, accelerometer, 6 minutes walk test

## Abstract

**Background:**

Cognitive impairment is common in patients with multiple sclerosis (MS). Physical activity is clearly linked to cognitive performance, and several studies have shown the importance of regular cognition testing, but such testing is still not routinely performed in clinical practice.

**Objective:**

This study aimed to investigate the association between cognition, disability, and physical performance in relapsing-remitting MS (RRMS) patients.

**Methods:**

A total of 41 patients with RRMS with an Expanded Disability Status Scale (EDSS) level of 0-5.5 and 20 healthy controls completed the MS Functional Composite (MSFC) test and the Symbol Digit Modality Test (SDMT). Six-Minute Walk (6MW) was evaluated for all participants, and they used an accelerometer for seven days.

**Results:**

A significant correlation was found between cognition and disability level measured by the MSFC (MSFC/SDMT, r = 0.668, *P* = .001) and between disability and 6MW (EDSS/6MW, r = −0.516, *P* = .001; MSFC/6MW, r = 0.348, *P* = .028) in the patients’ group. Cognition results (SDMT) were statistically significantly weaker in patients with EDSS >2.5 vs EDSS ≤2.5 or control group. Total daily activity (MVPS) correlated with cognition as measured by the SDMT in the control group but not in the patients’ group. In the EDSShigh group, better results on the 6MW test were associated with better cognition results as measured by the SDMT (r = 0.505, *P* = .039).

**Conclusion:**

There was a clear association between disability, 6MW and cognition. Better results on the 6MW predicted better cognition and disability.

Clinical trial registration number: NCT04115930

## Introduction

Cognitive impairment is very common in multiple sclerosis (MS), and 40-70% of patients have cognitive symptoms.^1,2^ These symptoms may be present in the early stages of the disease and even before the actual diagnosis of MS – that is, in the clinically isolated syndrome (CIS) stage – with a prevalence of 27-57%.^2^ The most common cognitive symptoms are a decline in information processing capacity and memory difficulties.^3,4^ Furthermore, the incidence of cognitive problems increases according to disease time, type, and progression, for example between relapsing-remitting MS (RRMS), secondary progressive MS (SPMS), and primary progressive MS (PPMS), so that the more progressive diseases (SPMS and PPMS) are associated with more severe cognitive symptoms than RRMS.^1,2,5-7^ Cognitive symptoms are usually also related to other symptoms, such as fatigue and relapses.^4^

Cognitive symptoms have a strong impact on patients’ daily functioning and quality of life. In several previous studies, the importance of regular cognition testing has been mentioned, but cognition is still not routinely assessed in MS patients.^4^ Cognitive symptoms are examined using different kind of methods, including the Symbol Digit Modality Test (SDMT), the Paced Auditory Serial Addition Test (PASAT), the Brief International Cognitive Assessment for MS (BICAMS), and full neuropsychological assessment. Regular follow-up is helpful in evaluating disease activity and treatment efficacy. Medication can have only very limited effects on cognitive symptoms, but cognitive rehabilitation can positively affect long-term patient outcomes. Cognitive rehabilitation has been shown to be beneficial: for example, in addition to improving patients’ ability to perform everyday activities, it reduced the risk of dementia by 29% in the 10 years after rehabilitation.^7^ There is evidence of the effects of various types of physical training, including aerobic training, resistance training, balance training, and various motor and neurophysiological exercises on MS and cognition.^7^ Previous studies have also shown that moderate-to-vigorous physical activity (MVPA) has a statistically significant correlation with cognition (SDMT) in MS patients.^8^

A full understanding of the association between cognitive performance and physical disability is still lacking especially at different levels of patients’ disability as measured by the EDSS. There is also a need for comprehensive but easily administered assessments for monitoring MS patients in clinical practice. The objective of this prospective cross-sectional study was to investigate the relationship between disability, cognition and physical performance including accelerometer-measured daily physical activity.

## Materials and Methods

### Recruitment, Patient Selection, and Control Group

The details of the study design and inclusion/exclusion criteria have been published previously.^9^ Patients were recruited between January 2016 and May 2018 through four MS patient associations. A total of 172 patients responded to the web-based screening questionnaire, including 112 patients with RRMS, 28 with SPMS, and 29 with PPMS. In total, 94 patients met the inclusion criteria, of whom 41 were willing to participate.

The inclusion criteria for this study were diagnosed RRMS, aged 18-55 years, and an Expanded Disability Status Scale (EDSS) level between 0 and 5.5. The exclusion criteria were relapse in the previous month, progressive disease (SPMS/PPMS), and diseases or injuries that significantly affect physical activity level. Disability level was measured using the EDSS (mean 2.75, median 2.5). The patients were divided into two groups based on their EDSS level (EDSSlow ≤2.5 [n = 22] and EDSShigh >2.5 [n = 18]). The control group comprised 20 healthy volunteers (employees and students) who were recruited from the University of Eastern Finland and the Savonia University of Applied Sciences.^9^

### Standard Protocol Approvals, Registration, and Patient Consent

The Research Ethics Committee of the Northern Savo Hospital District (reference 411/13.02.00/2015) approved this study (01/19/2016). The study was performed in accordance with the Declaration of Helsinki. Furthermore, the study was registered with Clinical Trials (https://www.clinicaltrials.gov, NCT04115930). The participants were provided with oral and written information about the study, and all provided written consent.^9^

### Data Collection and Clinical Assessments

The participants completed the questionnaires and physical tests on the same day and in the same order. The EDSS of the first five patients had to be administered on a different day, but this was completed within four weeks of the other tests’ completion. An EDSS value was not obtained from one patient. The same researcher conducted all the EDSS tests. On a test day, participants’ height (cm), weight (kg), and waist circumference (WC) were measured (Table 1).^9^

**Table 1.** Patient and Control Group Characteristics

Group	Age (y)	Height (cm)	Weight (kg)	BMI	Waist (cm)
Patient n = 41	44.6 ± 6.7***	166.7 ± 6.9	76.8 ± 14.7	27.7 ± 4.9*	93.1 ± 13.3**
EDSS-low n = 22	44.4 ± 7.4	165.2 ± 6.4	73.1 ± 13.0	26.8 ± 4.6	89.9 ± 13.1
EDSS-high n = 18	44.3 ± 5.9	168.8 ± 7.2	81.4 ± 16.2	28.6 ± 5.4	96.8 ± 13.2
Control n = 20	35.6 ± 8.7	170.0 ± 9.3	71.2 ± 14.5	24.6 ± 4.3	83.6 ± 10.9

Independent paired t test (mean/SD).

n = 41 (men 5%, women 95%).

n = 20 (men 15%, women 85%).

*P* < .001***, *P* < .01**, *P* < .05*, patients vs control.

The characteristics of the entire study population have been described previously.^9^

The MSFC test (T25-FW, 9-HPT, and PASAT-3), SDMT test, and Six-Minute Walk test (6MW) were administered by the same researcher. The Task Force dataset was used to produce composite scores on the MSFC test, which enable the comparison of results between different studies. The MSFC test has been shown to correlate with other indicators of disease in MS (validity) and to have high reliability. However, for the PASAT and MSFC, learning effects may need to be considered and monitored if the tests are repeated several times.^10^ All participants performed the SDMT. It has been shown that total SDMT scores of ≤55 correlate with impaired cognition, with a sensitivity of 0.82.^11^ A change in score of 3-4 points can be considered clinically meaningful. The 6MW was performed on a flat surface, and the same researcher supervised all test performances. Finally, participants were instructed to use the accelerometer (ActiGraphTM wGT3X-B; Pensacola, FL, USA) and exercise diary for seven days.

Using the thresholds provided by the manufacturer, accelerometer-measured physical activity was classified into five different activity levels: sedentary (SED), light (LPA), moderate, vigorous, and very vigorous physical activity. For the analysis, moderate-to-vigorous activity was classified as MVPA and light to very vigorous activity as MVPS. Participants had an average of seven valid measurement days (range: 6-8). For a measurement day to be considered valid, participants were required to have worn the accelerometer for more than 600 min/day.^9^

### Statistics

IBM SPSS version 29.0 (IBM, NY) was used for the statistical analyses. Data are as means ± SD ( standard deviations) and 95% CI (confidence intervals). All reported *P*-values are based on two-tailed statistical tests with the significance level set at *P* < .05. RRMS patients’ disability, cognition, physical activity, and physical performance scores were compared with those of the control group or different RRMS subgroups using independent paired t-tests. Correlation analyses were performed between EDSS, cognition, and physical activity using Pearson correlation tests. The strength of associations was determined by the Pearson correlation coefficient and classed as weak (0.10-0.39), moderate (0.40-0.69), strong (0.70-0.89), or very strong (0.90-1.00).^9^ Cohen D was used to determine the effects size and it is assessed as follows; small (d = 0.2), medium (d = 0.5) and large (d = 0.8).

### Data Availability

No identified patient data will be shared, and nor will any study-related documents. Anonymised data will be shared with qualified investigators on request.

## Results

Participants in the control group were younger (*P* < .001) and had lower body mass indices (BMI) and WCs than patients with RRMS (*P* = .020 and *P* = .008, respectively; Table 1). No statistically significant differences existed within the patients’ subgroups (EDSShigh and EDSSlow groups). Most participants in both the patient and control groups were women.

SDMT scores were significantly lower in the RRMS group than in the control group (*P* < .001, Cohen’s d = −0.96). Patients with an EDSS level of 0-2.5 (EDSSlow group) were found to have lower scores on the SDMT (*P* = .041) than healthy controls. Patients with an EDSS level of 3-5.5 (EDSShigh group) had even lower values than the EDSSlow group, and the difference in scores between the EDSShigh and control groups was statistically highly significant (*P* < .001). Furthermore, MSFC test scores were lower in the RRMS than the control group (*P* < .001, Cohen’s d = −0.99) and in the EDSShigh (*P* = .004) compared to the EDSSlow group. Moreover, PASAT scores were significantly lower in the RRMS group (*P* = .049, Cohen’s d = −0.55) than in the control group, as well as in the EDSShigh group compared to the EDSSlow group (*P* = .04) (Table 2).

**Table 2.** Disability and Cognition Results in Patient and Control Groups

Group	EDSS	MSFC	SDMT	PASAT
Patient n = 41	2.8 ± 1.1	0.3 ± 0.4^a***^	48.1 ± 9.4^a***^	44.2 ± 11.0^a*^
	CI [2.4, 3.1]	CI [0.19, 0,44] d = −0.99	CI [45.1, 51.1] d = −0.96	CI [40.7, 47.7] d = −0.55
EDSS-low n = 22	1.9 ± 0.6	0.5 ± 0.4	51.1± 9.3	47.8 ± 9.3
	CI [1.6, 2.2]	CI [0.33, 0.65]	CI [47.0, 55.2]	CI [43.7, 51.9]
EDSS-high n = 18	3.8 ± 0.6	0.1 ± 0.4^b**^	45.4 ± 8.1^b*^	40.8 ± 11.6^b*^
	CI [3.5, 4.1]	CI [−0.05, 0.031]	CI [41.4, 49.4]	CI [35.0, 46.5]
Control n = 20	—	0.7 ± 0.3	56.6 ± 7.2	49.9 ± 8.6
		CI [0.55, 0.83]	CI [53.2, 59.9]	CI [45.8, 53.9]

EDSS, expanded disability status scale; MSFC, multiple sclerosis functional composite; SDMT, symbol digit modality test; PASAT, paced auditory serial addition test; CI, confidence interval 95%; d, Cohen’s effect size [patients group vs control group, small (d = 0.2), medium (d = 0.5) and large (d = 0.8)].

Independent paired t test (mean/SD).

*P* < .001***, *P* < .01**, *P* < .05*, a patients group vs control group, b EDSSslow vs EDSShigh, *not measured from control group.

Table 3 presents the physical activity data across the four different categories (SED, LPA, MVPA, MVPS) measured by the accelerometer data, steps, and 6MW test performance. MVPA time, steps, and 6MW distance were significantly higher in the control group than in the RRMS group (*P* < .001, Cohen’s d = −1.02, *P* = .004, Cohen’s d = −0.85, *P* < .001, Cohen’s d = −1.3, respectively). No significant differences in any other values were found between these groups. There were also no significant differences between these values across the EDSSlow and EDSShigh groups. However, 6MW distance was significantly longer in the EDSSlow than the EDSShigh group (*P* < .001).

**Table 3.** Physical Activity and Physical Performance in Patient and Control Group

Group	SED (min)	LPA (min)	MVPA (min)	STEPS	MVPS (min)	6 MW(m)
Patient total n = 38	583 ± 109	167 ± 49	34 ± 20^a***^	6341 ± 2691^b**^	201 ± 59	620.8 ± 77.3^a***^
	CI [547.1, 618.6] d = −0.12	CI [150.3, 182.8] d = 0.01	CI [27.4, 40.5] d = −1.02	CI [5456, 7225] d = −0.85	CI [181.3, 219.8] d = −0.39	CI [596.0, 645.5] d = −1.3
EDSS-low n = 20	609 ± 87	167 ± 55	36 ± 20	6839 ± 2937	203 ± 66	655.2 ± 63.0^b***^ CI [627.3, 683.2]
	CI [567.8, 649.3]	CI [141.4, 192.6]	CI [26.7, 45.5]	CI [5464, 8213]	CI [172.3, 234.0]	
EDSS-high n = 17	553 ± 129	165 ± 45	32 ± 20	5803 ± 2417	197 ± 52	577.9 ± 75.6
	CI [486.6, 618.7]	CI [141.9, 188.6]	CI [21.4, 42.4]	CI [4559, 7045]	CI [170.4, 224.1]	CI [539.1, 616.8]
Control n = 19	595 ± 73	166 ± 39	54 ± 19	8557 ±2413	222 ± 49	723.0 ± 79.9
	CI [559.7, 630.4]	CI [150.3, 182.7]	CI [44.8, 63.0]	CI [7393, 9719]	CI [198.7, 246.3]	CI [685.6, 760.4]

SED, sedentary behaviour; LPA, light-intensity physical activity; MVPA, moderate-to-vigorous physical activity; STEPS, step count; MVPS, LPA + MVPA + very vigorous physical activity; 6MW, six-min walk; CI, Confidence Interval 95%.

d = Cohen´s effect size [patients group vs control group, small (d = 0.2), medium (d = 0.5) and large (d = 0.8)].

Independent paired t test (mean/SD) *P* < .001***, *P* < .01**, *P* < .05*, a patients group vs control group, b EDSSslow vs EDSShigh.

The accelerometer results have been described previously.^9^

### Associations Between Cognition and Disability

**Figure 1.**
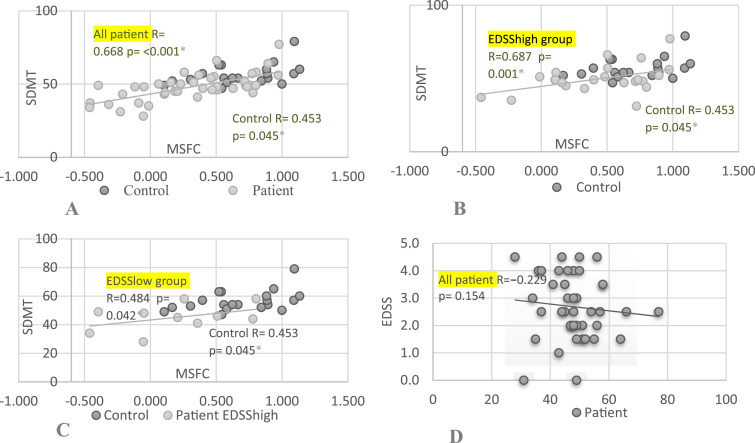
Associations Between Cognition and Disability of MS Patient and Controls. Abbreviations: SDMT, Symbol Digit Modality Test; MSFC, Multiple Sclerosis Functional Scale; EDSS, Expanded Disability Status Scale; Patient, all Patients; Control, Control Group; (A) SDMT Correlation to MSFC; (B) SDMT Correlation to MSFC in EDSShigh Group; (C) SDMT Correlation to MSFC in EDSSlow Group; (D) EDSS Correlation to SDMT. r = Pearson Correlation (r = [0.1-0.39] Weak, Moderate [0.40-0.69], Strong [0.7-0.89], or Very Strong [0.9-1.00]). p = Statistical Significance (*P* ≤ .05)^*^

Cognition (SDMT) correlated with disability (MSFC) in all RRMS groups and the control group (Figure 1). There was no correlation between EDSS and SDMT or PASAT.

### Associations Between Cognition and Physical Activity

**Figure 2.**
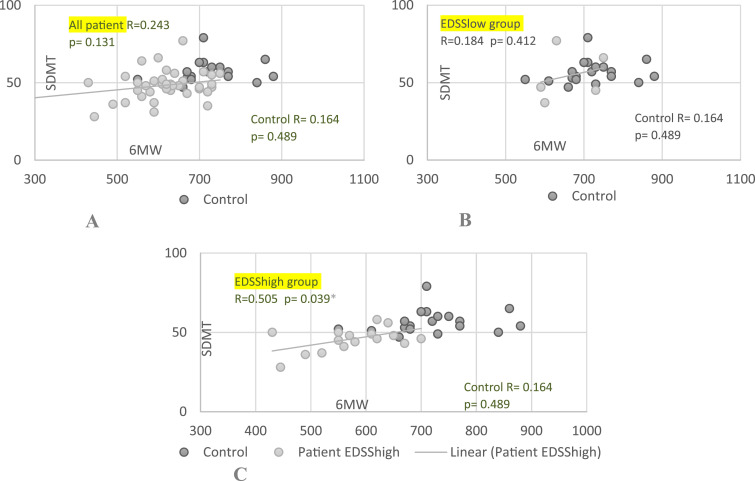
Associations Between Cognition and Physical Performance. Abbreviations: SDMT, Symbol Digit Modality Test; 6MW, Six-Min Walk Test; Patient, Patients Group; Control, Control Group; (A) SDMT Correlation to 6MW Whole Patient Group; (B) SDMT Correlation to 6MW in EDSSlow Group; (C) SDMT Correlation to 6MW in EDSShigh Group. r = Pearson Correlation (r = [0.1-0.39] Weak, Moderate [0.40-0.69], Strong [0.7-0.89], or Very Strong [0.9-1.00]). p = Statistical Significance (*P* ≤ .05)^*^

SDMT and 6MW performance were correlated in the EDSShigh group (r = 0.505, *P* = .039) (Figure 2). There were no correlation in any other groups. SDMT correlated with sedentary time (SED, r = 0.456, *P* = .05), moderate-to-vigorous physical activity (MVPS, r = −0.456, *P* = .05), and MVPA (r = −0.483, *P* = .036) in the control group but not in the RRMS group.

### Associations Between Physical Activity and Disability

**Figure 3.**
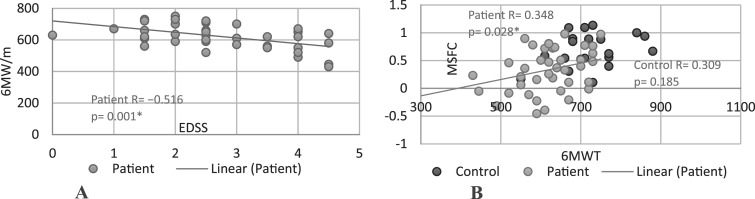
Associations Between Physical Performance and Disability of MS Patient and Controls. Abbreviations: EDSS, Expanded Disability Status Scale; 6MW, Six-Min Walk Test; MSFC, Multiple Sclerosis Functional Composite; Patient, Patients Group; Control, Control Group; (A) EDSS Correlation to 6MW; (B) MSFC Correlation to 6MW. r = Pearson Correlation (r = [0.1-0.39] Weak, Moderate [0.40-0.69], Strong [0.7-0.89], or Very Strong [0.9-1.00]). p = Statistical Significance (*P* ≤ .05)^*^

The 6MW test correlate with disability (both EDSS and MSFC) in the RRMS group (Figure 3).The correlation was a moderate between the EDSS and 6MW (r = − 0.516, *P* < .001 and weak between the MSFC and the 6MW (r = 0.348, *P* = .028). No correlations were found in any other of the groups.

## Discussion

This study revealed a clear correlation between disability levels (measured by the MSFC) and cognition (measured by the SDMT) in patients with RRMS. The differences in cognition (SDMT) and functional capacity (MSFC) between the control and patient groups were statistically highly significant and large as measured by Cohen’s effect size. The cognitive capacity measured through SDMT and PASAT tests showed differences amongst the EDSS high, EDSS low and the control group. Cognitive capacity was weakest in the EDSS high group, slightly better in the EDSS low group, and highest in the control group. The difference between EDSS high and EDSS low scores in SDMT was also clinically significant reflecting that poorer cognitive function in patients which have greater EDSS score. The results are logical, and some previous studies support these findings. Borreli et al found that lower SDMT scores were associated with higher EDSS score and psychological dimension of fatigue symptoms.^12^ Va´zquez-Marrufo et al found remarkable correlation between moderate score of SDMT and EDSS.^13^ However, in this study, there was no direct correlation between the whole group’s EDSS values and cognition as measured by SDMT or PASAT. This result also aligns with the findings of some previous studies, which have shown that EDSS does not objectively reflect cognitive impairment.^14^ However, some studies have found an association between EDSS and cognition using other neuropsychological batteries.^15^

There are several studies on disability, cognition, and physical activity.^16,17^ Exercise and physical activity have been shown to have positive effects on cognition measured by SDMT. Furthermore, average changes in daily step counts were also associated with changes in 6MW regardless of disability level.^18^ However, there is insufficient evidence on the effect of exercises, physical activity and physical condition on effective improvement of cognition.^16^ There is no comprehensive article that has used EDSS as a measure of functional capacity, while simultaneously assessing physical activity with accelerometer, cognition with SDMT, and physical performance with 6MW. This study has also used the MSFC, which increases the coverage of the assessment of disability and cognition. In this study, we found a clear association between disability measured by MSFC, cognition measured by SDMT, and 6MW in RRMS patients. There was also correlation with 6MW, and disability measured by EDSS in RRMS patients but not in the control group. The difference in physical performance (6MW) between the control and patient groups was statistically highly significant and large as measured by Cohen’s effect size. A shorter walking test result predicts poorer functional capacity. The 6MW correlate also cognition in more severe patients’ group when EDSS was more than 2.5. The weaker results in 6MW reflect the weaker cognition measured by SDMT. With better functional capacity (low EDSS), patients had better condition and better results in cognitive tests. However, MVPA, which measured physical activity, did not correlate with cognition, although it had a statistically significant relationship with patients’ physical performance (6MW). The physical activity level did not differ statistically between the groups except for MVPA and steps between the control and patient groups. The differences were statistically highly significant and large as measured by Cohen’s effect size. Patients with better functional capacity were slightly more active, eg, engaging in more moderate-intensity exercise and regarding daily step count. In practice, patients with worse functional ability had worse results in the cognitive test and their 6-minute walk test result was also weaker.

Manglani et al^19^ (2023) found a connection between working memory /processing speed and average kilocalorie consumption, moderate-to-vigorous physical activity (MVPA), average daily number of steps, and gender (higher scores in males). In this study, we did not find a correlation between cognition and physical activity measured by accelerometer. The reason for this was probably too small a sample size in different patient groups and too short a follow-up time with an accelerometer. Further studies are needed. However, in the controls, we did find a correlation between moderate-to-vigorous physical activity and MVPS and cognition measured by SDMT. Higher activity level was associated with higher SDMT scores. Similar findings were obtained in a previous study.^8^

The SDMT has been found to be one of the most valid and easily administered tests for measuring MS-related cognitive dysfunction and has high sensitivity and specificity.^20^ It is also slightly more sensitive than the PASAT for measuring cognitive impairment.^21^ It is currently recognised as the gold standard for rapid cognitive screening in MS.^4,22,23^ The SDMT focuses on processing speed assessment but is not specific, for example, to learning and memory. However, it has also been shown to correlate with several different measures, including performance, fatigue, brain atrophy, and EDSS.^4,22^ The SDMT is also part of the neuropsychological battery referred to as the ‘Brief International Cognitive Assessment for MS’ (BICAMS). The SDMT has been proposed as a replacement for the PASAT because it is easier and faster to administer and does not correlate strongly with psychological stress.^3,4^

A strength of this study is the wide range of measurements obtained and the fact that the same researcher performed the majority of the tests. The same person performed the EDSS examination for all patients. Two tests were used to measure disability (EDSS and MSFC) and cognition (PASAT and SDMT). It must be noted that MSFC includes PASAT, to which SDMT clearly correlates. However, also in the other subtests of the MSFC (9-HPT and 25FWT) were correlated in the SDMT patient group, but not in the control group. The weaknesses of the study are the rather small number of patients and relatively short duration of the accelerometer measurement. The control group was younger compared to the patient group. This should be taken into account, although in a study of this type and in this age group it is not expected to have a significant impact on the results. The lack of longitudinal data may also partially limit the assessment of causality between cognition, disability, and physical performance. The limited sample size may be affecting the generalizability of the findings. The correlation results were supported by several different measurement methods and may partially eliminate the effects of confounding variables. However, when analysing the research results, it is important to carefully consider which measures were used and what possible factors may have influenced the results. Longer-term monitoring of physical activity would improve the reliability of the study and could enable identification of more differences between the patient and control groups. Recruiting a larger group of patients would enable more detailed subgroup analyses in the different disability categories and thus provide more reliable information on disability, cognition and physical activity.

## Conclusion

This study found a clear association between disability and cognition and physical performance in RRMS patients. The study further emphasised the importance of SDMT in the assessment of cognition and the importance of the MSFC in the assessment of disability in clinical practice. The MSFC and the SDMT complement each other well in assessing patients’ disability and cognition in clinical use. Physical condition measured at 6MW correlates well with MS patients’ disability and with cognition in patients with EDSS 3-5.5. The measurement is easy to perform and based on this study, its use is recommended when investigating the connection between the aforementioned issues. Further information is needed on cognition, disability and functional ability in different types of MS patients.

## Data Availability

No identified patient data will be shared, and nor will any study-related documents. Anonymised data will be shared with qualified investigators on request.*

## Appendix

### Abbreviations

SDMT: Symbol digit modality test
MSFC: Multiple sclerosis functional composite
PASAT: Paced auditory serial addition test
SED: Sedentary time
LPA: Light physical activity
MVPA: Moderate-to-vigorous physical activity
MVPS: Total physical activity
6MW: Six-minute walk.
